# Dosimetric Evaluation and Clinical Application of Radioactive Iodine-125 Brachytherapy Stent in the Treatment of Malignant Esophageal Obstruction

**DOI:** 10.3389/fonc.2022.856402

**Published:** 2022-03-24

**Authors:** Zhe Ji, Qianqian Yuan, Lei Lin, Chao Xing, Xusheng Zhang, Sen Yang, Yuliang Jiang, Haitao Sun, Kaixian Zhang, Junjie Wang

**Affiliations:** ^1^Department of Radiation Oncology, Peking University Third Hospital, Beijing, China; ^2^Department of Oncology, Tengzhou Central People’s Hospital, Zaozhuang, China

**Keywords:** radioactive iodine-125 seed, stent, esophageal cancer, dosimetry, efficacy

## Abstract

**Objective:**

To evaluate the dosimetric characteristics and the clinical application of radioactive iodine-125 brachytherapy stent (RIBS) in malignant esophageal obstruction.

**Methods:**

The dose distribution of RIBS with different seed spacing, diameter and length was studied by treatment planning system (TPS) calculation, thermoluminescence dosimeter (TLD) measurement and Monte Carlo (MC) data fitting. And the data of esophageal cancer patients who were treat with this type of RIBS was analyzed retrospectively.

**Results:**

Doses around the RIBS calculated by the TPS lay between those measured by the TLDs and those simulated by the MC, and the differences between the three methods were significant (p<0.05), the overall absolute dose differences among the three methods were small. Dose coverage at 1.5 cm from the center was comprehensive when the activity reached 0.6 mCi. Both the conformability and the uniformity of isodose lines produced by a seed spacing of 1.0 cm were superior to those produced by a seed spacing of 1.5 cm. The data of 50 patients treated with RIBS was analyzed. They were followed up until February 2020 when all of the patients died. The overall improvement rate of dysphagia after RIBS implant was 90%. Moderate and severe complications with an incidence of more than 10% were hematemesis (28%), pain (20%), and lung infection (10%). Stent restenosis occurred in 4 patients at a median interval of 108 days from the procedure. The overall incidence of fatal complications was 38% (including hematemesis, infection and asphyxia). The median survival time of patients with and without a history of radiotherapy were 3.4 months and 6 months, respectively, the difference of which was significant (p=0.021). No other factors affecting survival were identified. For patients with and without a history of radiotherapy, the incidences of fatal complications were 51.7% and 19%, respectively (p=0.019). No correlation between dose and stent restenosis was found.

**Conclusion:**

TPS calculations are suitable for clinical applications. RIBS can effectively alleviate obstructive symptoms for patients with malignant esophageal obstruction, but the incidence of fatal complications was high, care should be taken when choosing this treatment.

## Introduction

Esophageal cancer is common and accounts for 572,000 new cases and 505,000 deaths each year, ranking the 9th most common type and the 6th leading cause of death among all cancers (1). While esophageal cancer is managed mainly by surgery and chemoradiotherapy (2), it is often more important to manage the problem of obstruction among patients with severe dysphagia. However, not only it is difficult for these patients to tolerate surgery or chemoradiotherapy due to unfavorable factors, such as advanced age, poor general condition, low body mass index, and severe comorbidities, but symptoms of obstruction also cannot be rapidly alleviated because of the delayed response of tumor for chemoradiotherapy.

The application of metal stents in the treatment of malignant esophageal obstruction was first reported by Frimberger (3) in 1983. Owing to its accurate positioning and simple operation, this technique can quickly and effectively relieve symptoms of dysphagia as well as improve patients’ nutritional status and quality of life, making it a standard method in treating malignant esophageal obstruction (4).

As conventional esophageal stents expand the esophagus simply by mechanical forces, they demonstrate no therapeutic effect on tumors that have caused the stenosis. In addition, due to growth of the tumor or proliferation of the granulation tissue, approximately 30–40% patients experience restenosis after stent implant (5), compromising the long-term efficacy of the stent. This issue has been resolved with the use of radioactive iodine-125 brachytherapy stent (RIBS), which can expand the esophagus while simultaneously performing brachytherapy on the tumor, thereby achieving the dual purpose of relieving dysphagia as well as eliminating cancer (6, 7). A recent randomized study showed that RIBS can prolong patient survival more effectively than conventional stents (8).

At present, there are few studies on the dosimetry of RIBS. In addition, existing studies are inconsistent regarding methods on the bundling methods and the quantity of seeds and provided little information on the prescription dose. The purpose of this study was to evaluate the dosimetric characteristics and the clinical application of RIBS in treating malignant esophageal obstruction, thereby providing references for its clinical implementation.

## Materials and Methods

### RIBS Dosimetric Study

#### Materials

Several materials were used in this study, including a covered self-expanding esophageal stent (Micro-Tech (Nanjing) Co., Ltd., Jiangsu Province, China), and iodine-125 (I-125) seeds, type 6711_1985, with an outer diameter of 0.8 ± 0.02 mm and a length of 4.5 ± 0.2 mm (Atomic High Technology Co., Ltd., Beijing, China). The seed had an outer shell of titanium, a half-life of 59.4 days, a dose-rate constant of 0.965 cGy/(h∙U), and various activities of 0.5, 0.6, 0.7, 0.8, 0.9, and 1.0 mCi. The following materials were also used: Allura Xper FD20 (Philips, Amsterdam, Netherlands), a digital subtraction angiography (DSA) system; OLYMPUS GIF-H290 (Olympus Co., Ltd., Tokyo, Japan), a gastroscope; paraffin as an analytical purity (Tianjin Basifu Chemical Co., Ltd., Tianjin, China); Brilliant Big Bore (Philips, MA, USA), a CT simulator; and a treatment planning system (TPS) (Beijing Feitianzhaoye Technology Co., Ltd., Beijing, China). Dose calculations at different distances to the radioactive source in the TPS were based on the American Association of Physicists in Medicine (AAPM) TG43 report and its updated documents (9, 10) and on thermoluminescence dosimeters (TLDs) (Beijing Institute of Chemical Defense, Beijing, China). These dosimeters were made of TLD-200 (LiF: Mg, Cu, P) square chips (3.2 cm × 3.2 cm) with a measurement range of 10 µGy to 10 Gy and a detection limit of 0.1 µGy, and a TLD reader, Harshaw 3500 (Thermo Fisher Scientific, MA, USA).

#### Methods

##### Preparation of Different Specifications of RIBS (4 Specifications)

**Figure 1:**
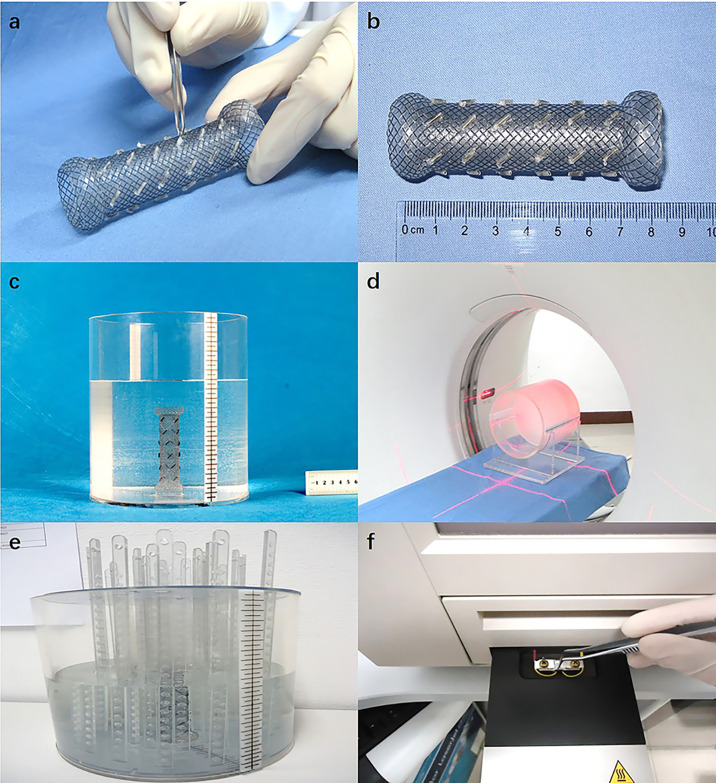
**(A)** The periphery of the stent was bundled with a polyurethane catheter as the delivery sheath, which contains radioactive I-125 seeds; **(B)** The complete RIBS; **(C)** The RIBS was fixed in a cylindrical phantom filled with paraffin melt; **(D)** CT scan (images were exported to the TPS); **(E)** After the RIBS was fixed to the center of the cylindrical phantom and filled with distilled water, the TLD measuring rods were placed at different distances to the center of the stent; **(F)** TLD were read out 24 h after irradiation.

Specification 1: length = 8.0 cm, diameter = 2.0 cm, and seed spacing = 1.0 cm; Specification 2: length = 8.0 cm, diameter = 2.0 cm, and seed spacing = 1.5 cm; Specification 3: length = 8.0 cm, diameter = 1.3 cm, and seed spacing = 1.0 cm; Specification 4: length = 12.0 cm, diameter = 2.0 cm, and seed spacing = 1.0 cm. The delivery sheath was made of polyurethane (synthesized from polytetrahydrofuran ether glycol, 4,4’-diphenylmethane diisocyanate, and 1,4-butanediol; density = 1.19 g/cm3). The RIBS was subsequently created by bundling a conventional stent with the delivery sheath containing radioactive I-125 seeds (**Figures 1A, B****)**.

##### TPS Calculations

The RIBS was vertically fixed in a cylindrical Perspex phantom (thickness = 0.8 cm, diameter = 20 cm, and height = 20 cm) filled with solid paraffin melt. Once the paraffin was cooled and solidified, the phantom was scanned using computed tomography (CT) (window width = 300 HU, window level = 15 HU, and slice thickness = 5 mm) (**Figures 1C, D****)**, and images were exported to the TPS. Subsequently, with the center of the stent as the origin, cumulative doses at a distance of 1.5 cm, 2.0 cm, 2.5 cm, 3.0 cm, 3.5 cm, 4.0 cm, and 5.0 cm from the origin were calculated at both 0° and 90°, the average of which was computed as the result.

##### TLD Measurements

The RIBS was fixed in the center of the cylindrical Perspex phantom and was filled with distilled water. Subsequently, with the center of the stent as the origin, TLD measuring rods were placed at 1.5 cm, 2.0 cm, 2.5 cm, 3.0 cm, 3.5 cm, 4.0 cm, and 5.0 cm from the origin. After being irradiated for 24 h, TLDs were removed from the phantom and read out in the TLD reader (**Figures 1E, F****)**, with the average value at 0° and 90° calculated as the result.

##### Monte Carlo (MC) Simulation

An actual geometry source model was established using the MC N-Particle Transport Code (MCNP) based on the radioactive I-125 seed model (6711), so as to calculate the dose distribution of the radioactive stent in a calculation range of 1 keV to 100 keV (9, 11). In the simulations, photo-electric effect, compton scattering, rayleigh scattering and pair production were considered for photons. The energy cut-off value for photons was set to the minimum value 0.25 keV. Secondary electrons were not tracked assuming that all of their energy is deposited at the location of their generation. The number of events in each simulation was set as 1x10^8 to achieve an average statistical uncertainty less than 1%. To validate the accuracy of seed modeling and MC simulation, the dose distribution of single I-125 seed was simulated and the radial dose and anisotropy functions were calculated and compared with reference data (12, 13). The radial dose function g(r) from 0.5 to 100 mm and the 2D anisotropy functions at 5, 10, 15 and 20 mm for the polar angle 0°≤ θ ≤ 90◦ were extracted from the calculated dose distribution and found in good agreement with the relative differences being less than 2.0%. This confirms that the seed model and the MC simulation of this study are valid. The model was first placed in the center of a sphere with a radius of 10 cm and water as a medium. Subsequently, with the center of the stent as the origin, doses at a distance of 1.5 cm, 2.0 cm, 2.5 cm, 3.0 cm, 3.5 cm, 4.0 cm, and 5.0 cm from the origin were calculated at both 0° and 90°, the average of which was computed as the result.

### Clinical Outcomes of RIBS in the Treatment of Esophageal Cancer

#### Patient Information

We retrospectively analyzed the data of patients with malignant esophageal obstruction treated with this type of RIBS from July 2014 to November 2019. The criteria of RIBS treatment were: ① Patient’s age ≥ 18 years; ② Diagnosis of esophageal cancer was pathologically confirmed; ③ Patient experienced Grade 3–4 dysphagia according to the criteria proposed by the Cardiovascular and Interventional Radiological Society of Europe (CIRSE) (14), which stated that Grade 0 = normal diet, Grade 1 = soft food only, Grade 2 = semi-solids only, Grade 3 = liquids only, and Grade 4 = complete dysphagia; ④ Patient could not tolerate surgery or chemoradiotherapy due to extensive tumor growth, metastasis, or poor medical conditions; ⑤ Patient demonstrated clear consciousness as well as good compliance and was cooperative with treatment; and ⑥ Patient had a Karnofsky Performance Score (KPS) ≥ 60 and could tolerate treatment. The exclusion criteria were: ① The upper boundary of the lesion exceeded the seventh cervical vertebra; ② Patient had ulcerative esophageal cancer or esophageal fistula; ③ Patient suffered from Class II or higher bone marrow suppression and/or coagulation dysfunction; and ④ Patient had other contradictions such as severe cardiopulmonary insufficiency and liver and kidney insufficiency.

#### Treatment Methods

##### Preoperative Planning

Prior to the implant, a 5 mm enhanced CT scan of the lesion in all areas was acquired, and CT images were exported to the TPS to contour the esophageal lesions. Subsequently, a preoperative plan was created with various seed activities ranging between 0.4 and 0.8 mCi and prescriptions ranging between 60 and 80 Gy. Seeds were ordered once the total number and the activity were determined.

##### Production of the I-125 Seed Stent

A covered esophageal stent with a diameter of 1.8 cm or 2.0 cm was selected according to the length and stenosis of the patient’s esophageal lesions. Each layer of the stent was bundled with 5–6 seeds, and the layers were separated by 1.0 cm. Seed spacing was set according to the preoperative plan to ensure that the prescription was fulfilled. Once radioactive seeds were fixed on the periphery of the stent, the internal stent was inserted into the stent pusher catheter.

##### Stent Implant

The patient was placed in the lateral position, anesthetized at the oropharynx with lidocaine spray, and a bite block was placed in the mouth. Subsequently, after a radiographic guide wire and a catheter were inserted through the oral cavity, contrast agents were injected in the upper and lower ends of the lesion to display the extent and the degree of stenosis. The imaging guide wire was then replaced with a super-hard and super-long guide wire, while the catheter was removed. Next, a covered stent of the right size was selected according to the extent of the lesion and implanted together with the pusher along the super-hard wire. Once its position was verified using proximal positioning, the stent was released. It was required that the upper and lower ends of the inserted stent should exceed the lesion by at least 20 mm.

##### Postoperative Plan Verification

At 48–72 hours after stent implantation, a 5 mm CT scan was acquired for review. After images were sent to the TPS, the esophageal lesion was contoured, and seeds were identified in the system to calculate the actual dose delivered to the tumor target (D90; i.e., the dose received by 90% of the target volume).

#### Outcome Indicators

Primary outcome indicators of this study included relief of patients’ clinical symptoms and relevant complications. Complications were graded according to Common Terminology Criteria for Adverse Events (CTCAE) version 5.0 (15), there were five grades, as follows: mild/grade 1 (no symptoms and no treatment required), moderate/grade 2 (symptoms present and treatment required), severe/grade 3 (symptoms not controlled by drugs, and instrumentation or invasive procedure required), life-threatening/grade 4 (emergency treatment required), and death/grade 5. Secondary outcome indicators included patients’ survival. Factors influencing patients’ complications and survival were also investigated.

### Statistical Analysis

Data analysis was performed using the SPSS 20 statistical software (IBM, Armonk, New York, USA). Measurement data were expressed as mean ± standard deviation (X ® ± s), while count data were expressed as absolute value and percentage (rate). Comparisons of means and rates were conducted *via* the t-test and the chi-square test, respectively. Patient’s survival was calculated using the Kaplan-Meier method. Univariate analysis was performed using the log-rank test. The hazard ratio was derived *via* Cox regression. P < 0.05 was considered statistically significant.

## Results

### Dosimetric Results

#### TPS Method

The dose around the RIBS dropped rapidly with increasing distance from the origin. According to the TPS, for stents of various specifications, the average doses (Gy) at seven different locations from the origin (1.5 cm, 2.0 cm, 2.5 cm, 3.0 cm, 3.5 cm, 4.0 cm, and 5.0 cm) were 112.3 ± 31.50, 68.0 ± 20.71, 42.5 ± 16.28, 26.8 ± 9.95, 18.4 ± 7.18, 12.7 ± 5.76, and 7.8 ± 4.95, respectively, the differences of which were statistically significant (P < 0.001).

It was also noted that the dose increased linearly with increasing seed activity. For the six seed activities (0.5 mCi, 0.6 mCi, 0.7 mCi, 0.8 mCi, 0.9 mCi, and 1.0 mCi) investigated in the study, the average doses (Gy) at different locations from the origin were 26.4 ± 24.99, 32.3 ± 30.01, 38.6 ± 35.81, 45.4 ± 41.69, 50.9 ± 46.06, and 57.8 ± 50.67, respectively, differences of which were statistically significant (P < 0.001). The average doses (Gy) at 1.5 cm from the origin were 76.8 ± 14.55, 93.0 ± 18.55, 109.5 ± 22.41, 126.8 ± 28.29, 141.3 ± 27.39, and 155.8 ± 30.23, respectively, for the different seed activities. The dose coverage at 1.5 cm from the origin was comprehensive when the seed activity reached 0.6 mCi.

**Figure 2:**
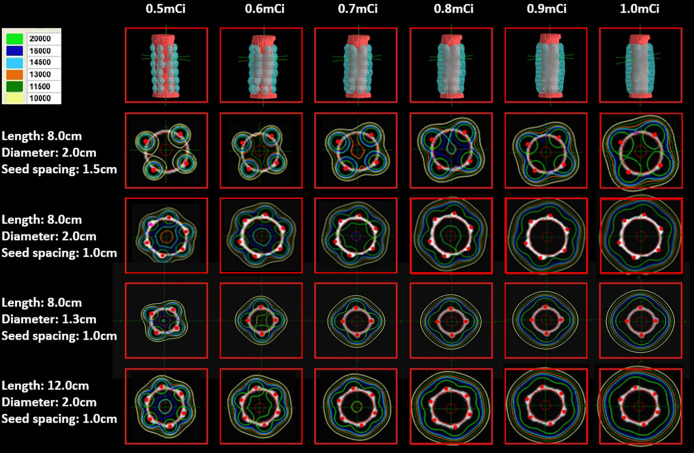
The higher the seed activity, the higher the dose. Both the conformability and the uniformity of the isodose lines produced by a seed spacing of 1.0 cm were superior to those produced by a seed spacing of 1.5 cm, the latter also showed a few dosimetric “cold spots”. The dose coverage was good with seed spacing of 1.0 cm and seed activity ≥ 0.6 mCi.

In addition, it was suggested that both seed spacing and stent length demonstrated significant effects on the dosimetry. The dose of the RIBS with a seed spacing of 1.5 cm was lower than that of the RIBS with a seed spacing of 1.0 cm. For the four different specifications of RIBS (Specification 1, Specification 2, Specification 3, and Specification 4), the average doses (Gy) at different locations from the origin were 30.6 ± 28.63, 41.0 ± 41.55, 43.6 ± 40.31, and 52.3 ± 46.22, respectively, the differences of which were statistically significant (P < 0.001). In addition, for the RIBS with a seed spacing of 1.0 cm, it was found that both the conformability and the uniformity of its isodose lines were superior to those of the RIBS with the seed spacing of 1.5 cm, whereas the latter showed several dosimetric “cold spots” on the plan (**Figure 2**).

#### TLD Method

**Figure 3:**
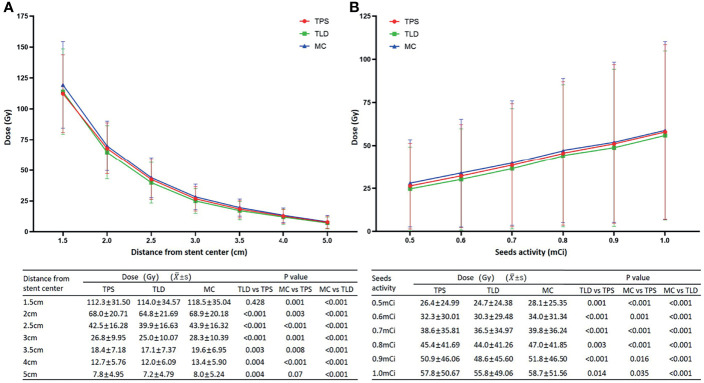
**(A)** Although the dose at 1.5 cm measured by the TLD was not significantly different from that calculated by the TPS, at all other locations, the TLD measurements were significantly lower. Alternatively, while the dose at 5.0 cm simulated by MC was not significantly different to that calculated by the TPS, all of the other MC simulation results were significantly higher. When compared to the TLD measurements, MC simulations were consistently higher with significant differences. **(B)** Dose increased linearly with increasing activity. Doses from the MC simulations were the highest, followed by TPS calculations and TLD measurements, and the differences between the three methods were significant.

The trends of TLD measurements were consistent with that of the TPS calculations. According to TLD measurements, for stents of various specifications, the average doses at seven different locations from the origin (1.5 cm, 2.0 cm, 2.5 cm, 3.0 cm, 3.5 cm, 4.0 cm, and 5.0 cm) were 114.0 ± 34.57, 64.8 ± 21.69, 39.9 ± 16.63, 25.0 ± 10.07, 17.1 ± 7.37, 12.0 ± 6.09, and 7.2 ± 4.79, respectively, the differences of which were statistically significant (P < 0.001). In addition, although the dose at 1.5 cm measured by the TLD was not significantly different to that calculated by the TPS (t = −0.807, P = 0. 428), at all other locations, TLD measurements were significantly lower than TPS calculations (t = 5.588, 4.881, 4.051, 3.358, 3.205, and 3.245, respectively, and P < 0.001, < 0.001, < 0.001, = 0.003, = 0.004 and = 0.004, respectively) (**Figure 3A**).

For the 6 seed activities (0.5 mCi, 0.6 mCi, 0.7 mCi, 0.8 mCi 0.9 mCi, and 1.0 mCi), the average doses (Gy) at different locations from the origin were 24.7 ± 24.38, 30.3 ± 29.48, 36.5 ± 34.97, 44.0 ± 41.26, 48.6 ± 45.60, and 55.8 ± 49.06, respectively, the differences of which were statistically significant (P < 0.001) (**Figure 3B**). Alternatively, the average doses (Gy) at 1.5 cm from the origin were 74.5 ± 17.16, 89.8 ± 20.60, 106.3 ± 23.67, 125.8 ± 24.10, 138.8 ± 28.91, and 149.3 ± 29.41, respectively. Similarly, TLD measurements were consistently lower than TPS calculations, and the differences were statistically significant (t = 3.802, 4.615, 6.914, 3.300, 5.243, and 2.640, respectively, and P = 0.001, < 0.001, < 0.001, = 0.003, < 0.001, and = 0.014, respectively).

For the four different specifications of RIBS (Specification 1, Specification 2, Specification 3, and Specification 4), the average doses (Gy) at different locations from the origin were 28.2 ± 27.88, 39.1 ± 40.42, 41.8 ± 39.36, and 50.8 ± 45.62, respectively, the differences of which were statistically significant (P < 0.001). Again, all TLD measurements were significantly lower than the TPS calculations (t = 5.835, 4.782, 4.106, and 4.836, respectively, and P < 0.001).

#### MC Method

The trends of MC simulations were consistent with that of the TLD measurements and TPS calculations. For stents of various specifications, the average doses (Gy) at seven different locations from the origin (1.5 cm, 2.0 cm, 2.5 cm, 3.0 cm, 3.5 cm, 4.0 cm, and 5.0 cm) were 119.5 ± 35.04, 69.9 ± 20.18, 43.9 ± 16.32, 28.3 ± 10.39, 19.6 ± 6.95, 13.4 ± 5.90, and 8.0 ± 5.24, respectively, the differences of which were statistically significant (P < 0.001). Although the dose at 5.0 cm simulated by MC was not considerably different from that calculated by the TPS (t = −1.904, P = 0.07), at all other locations, MC simulation results were significantly higher (t = −3.686, −3.347, −4.303, −3.800, −2.930, and −4.303, respectively, and P = 0.001, = 0.003, < 0.001, = 0.001, = 0.008, and < 0.001, respectively). When compared to the TLD measurements, the MC simulations were consistently higher with significant differences (t = −8.150, −6.970, −5.901, −5.584, −4.532, −5.274, and −3.984, respectively, and P < 0.001) (**Figure 3A**).

For the six seed activities (0.5 mCi, 0.6 mCi, 0.7 mCi, 0.8 mCi, 0.9 mCi, and 1.0 mCi), the average doses (Gy) at different locations from the origin were 28.1 ± 25.35, 34.0 ± 31.34, 39.8 ± 36.24, 47.0 ± 41.85, 51.8 ± 46.50, and 58.7 ± 51.56, respectively, the differences of which were statistically significant (P < 0.001) (**Figure 3B**). Alternatively, the average doses (Gy) at 1.5 cm from the origin were 78.8 ± 14.80, 96.3 ± 18.87, 111.5 ± 22.41, 129.0 ± 26.60, 142.8 ± 28.02, and 158.5 ± 30.88, respectively. The MC simulation results were consistently higher than the TPS calculations and TLD measurements, and for both comparisons, differences were statistically significant (versus TPS: t = −5.383, −3.684, −4.322, −5.717, −2.564, and −2.223, respectively, and P < 0.001, = 0.001, < 0.001, < 0.001, = 0.016, and = 0.035, respectively; versus TLD: t = −5.953, −5.653, −6.805, −6.274, −5.412, and −3.623, respectively, and P < 0.001).

For the four different specifications of RIBS (Specification 1, Specification 2, Specification 3, and Specification 4), the average doses (Gy) at different locations from the origin were 31.5 ± 29.26, 42.9 ± 41.37, 45.10 ± 41.12, and 53.5 ± 46.91, respectively, the differences of which were statistically significant (P < 0.001). All of the MC simulation results were significantly higher than TPS calculations (t = −3.315, −6.517, −4.175, and −4.577, respectively, and P = 0.002, < 0.001, < 0.001, and < 0.001, respectively). Similarly, MC simulation results were consistently higher than TLD measurements with significant differences (t = −6.779, −8.235, −5.301, and −7.213, respectively, and P < 0.001). However, the absolute dose difference between the two groups was less than 5 Gy. While the dose deviations in the high-dose area (1.5 and 2 cm from the origin) were 3.7% and 5.9%, respectively, the maximum deviation was 11.7% at 3 cm from the origin.

### Clinical Results

#### Patient Information

A total of 50 patients were included in this study. Patients included were either ineligible or unwilling to receive radiotherapy, while stents were urgently needed to relieve their symptoms. Detailed patient information is listed in **Table 1**.

**Table 1:** Patients’ baseline status before treatment.

General information	N (50 cases)	%
Gender		
Male	33	66.0
Female	17	34.0
Age (years old)	mean 71 (range, 52-88)	
KPS	median 70 (60-90)	
Initial stage		
III	17	34.0
IV	33	66.0
Location of lesions		
Upper-thoracic	12	24.0
Middle-thoracic	27	54.0
Lower-thoracic	7	14.0
Anastomosis	4	8.0
Disease type		
Initial treatment	19	38.0
Recurrence after treatment	24	48.0
Progress after treatment	7	14.0
History of radiotherapy		
No	21	42.0
Yes	29	58.0
Degree of obstruction		
Liquid diet	33	66.0
Complete obstruction	17	34.0
Albumin before treatment (g/L)	mean 35.1 (24.4-42.9)	
Hemoglobin before treatment (g/L)	mean 120.6 (87-201)	

#### RIBS Implant

The RIBS implants were successful in the first attempt for all patients. The average operation time was approximately 15 min. Information on RIBS and related doses is listed in **Table 2**. After the implantation, patients were administered with 250 ml of hot milk to help expand the stent.

**Table 2:** RIBS parameters.

Parameters	Median (range)
Length of stent (cm)	10 (6-12)
Diameter of stent (cm)	2 (1.8-2)
Seed spacing (cm)	1
Number of seeds per layer	6 (5-6)
Number of layers of seeds	5 (2-8)
Seeds activity (mCi)	0.6 (0.4-0.8)
Postoperative D90 (Gy)	56.7 (18.9-113.3)

#### Obstruction Relief

Dysphagia was significantly improved in 90% of patients (45/50) after RIBS implant. For the 33 patients who could only eat a full-liquid diet before the procedure, 15 cases could eat soft foods, 13 cases could eat semi-liquid foods, and five remained on a liquid diet after the procedure, indicating an overall improvement rate of 85%. Alternatively, for the 17 patients with complete dysphagia before the procedure, 6 cases could eat soft foods, 9 cases could eat semi-solids, and 2 cases could eat liquids after the operation, indicating an overall improvement rate of 100%.

#### Complications

The incidences of pain, foreign body sensation, cough, nausea and vomiting, asphyxia, hematemesis, perforation, stent displacement, restenosis, and fever/pneumonia were 80%, 14%, 28%, 8%, 6%, 28%, 6%, 4%, 8%, and 6%, respectively. Stent restenosis occurred in four patients at a median interval of 108 days (31–196 days) from the procedure. No incidence of seed loss was observed. Details of the complications are shown in **Table 3**. Out of all of the patients, 3 cases suffered from asphyxia, all of which were fatal; 14 cases from hematemesis, 11 of which were fatal; 3 cases from perforation, all of which were fatal (2 cases due to hematemesis and 1 case due to lung infection); and 8 cases from fever or lung infection, 5 of which were fatal (with 1 case accompanied by perforation). Therefore, the overall incidence of fatal complications was 38% (19/50). Moderate and severe complications with an incidence of more than 10% were hematemesis (28%), pain (20%), and lung infection (10%).

**Table 3:** Patients’ complications.

Complications	N	%
Pain		
No	10	20
Mild	30	60
Moderate	7	14
Severe	3	6
Foreign body feeling		
None	43	86
Mild	6	12
Moderate	1	2
Cough		
None	36	72
Mild	11	22
Moderate	2	4
Severe	1	2
Nausea and vomiting		
None	46	92
Mild	1	2
Moderate	3	6
Asphyxia		
None	47	94
Death	3	6
Haematemesis		
None	36	72
Moderate	3	6
Death (with 2 cases of perforation)	11	22
Perforation		
None	47	94
Death	3	6
Stent displacement		
No	48	96
Yes	2	4
Restenosis		
No	46	92
Yes	4	8
Fever/pulmonary infection		
None	42	84
Mild/Moderate	3	6
Death (with 1 case of perforation)	5	10

#### Prognosis and Influencing Factors

**Figure 4:**
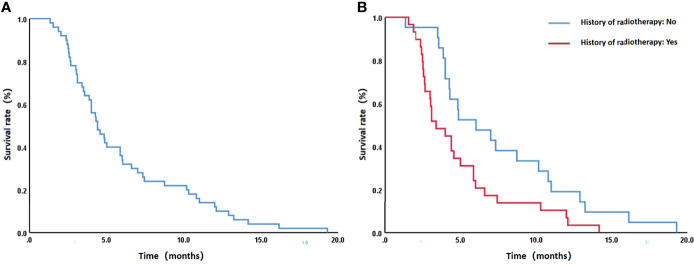
**(A)** The overall 6-month, 12-month, and 18-month survival rates of all patients were 34%, 12%, and 2%, respectively. **(B)** For patients with and without a previous history of radiotherapy, the median survival periods were 3.4 months (95% CIL 2.1–4.7 months) and 6 months (95% CI: 2.8–9.3 months), respectively, and the 18-month survival rates were 0% and 4.8%, respectively. The difference of the latter was statistically significant (P = 0.021).

**Figure 5:**
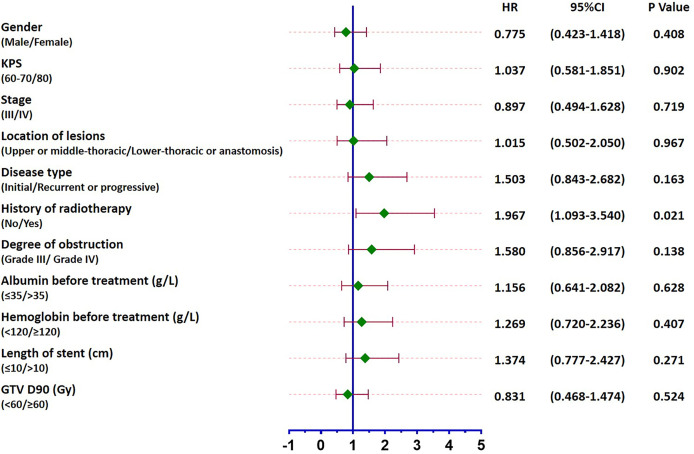
Forest plot of factors affecting patients’ survival. While a previous history of radiotherapy led to the prognosis of patients (P = 0.021), no other influencing factors were identified (P > 0.05).

Patients were followed up until February 2020 when all 50 patients died. The median survival was 4.4 months (95% CI: 3.4–5.4 months), and the 6-month, 12-month, and 18-month survival rates were 34%, 12%, and 2%, respectively (**Figure 4A**). A total of 6 patients survived for over 12 months, the longest of whom lived another 19.3 months after the procedure. Causes of death are listed in **Table 4**. The median survival periods of new and relapsed/uncontrolled patients were 5 months (95% CI: 2.9–9.1 months) and 3.9 months (95% CI: 2.8–4.9 months), respectively. The 18-month survival rates of the two groups were 5.3% and 3.2%, respectively, the difference of which was insignificant (P = 0.163). Alternatively, for patients with and without a previous history of radiotherapy, the median survival periods were 3.4 months (95% CIL 2.1–4.7 months) and 6 months (95% CI: 2.8–9.3 months), respectively. The 18-month survival rates of the two groups were 0% and 4.8%, respectively, which were significantly different (P = 0.021) (**Figure 4B**). Specific factors affecting patients’ survival are shown in **Figure 5**.

**Table 4:** Causes of death.

Cause of death	N	%
Tumor progression/cachexia	27	54
Hemorrhage of upper digestive tract	11	22
Infection	5	10
Asphyxia	3	6
Heart failure	1	2
Unknown reason	3	6

The incidences of fatal complications in new and relapsed/uncontrolled patients were 21.1% (4/19) and 48.4% (15/31), respectively, the difference of which was close to statistical significance (chi-squared = 3.736, P = 0.053). Alternatively, for patients with and without a previous history of radiotherapy, the incidences of fatal complications were 51.7% (15/29) and 19% (4/21), respectively, the difference of which was significant (chi-squared = 5.520, P = 0.019). More specifically, for patients whose interval between radiotherapy and stent implant was < 6 months or ≥ 6 months, the incidences of upper gastrointestinal bleeding were 62.5% (5/8) and 14.3% (8/29), respectively, the difference of which was significant (chi-squared = 6.741, P = 0.019).

No correlation was found between dose and survival. For patients whose D90 < 60 Gy or ≥ 60 Gy, the median survival periods were 4.3 months (95% CI: 3.6–5.0 months) and 4.6 months (95% CI: 3.0–6.1 months), respectively, while the 18-month survival rates were 3.7% and 0%, respectively, the difference of which was insignificant (P = 0.524) (**Figure 5**). Alternatively, the incidences of fatal complications of patients whose D90 ≤ 50Gy or > 50 Gy were 18.8% (3/16) and 47.1% (16/34), respectively, the difference of which was close to statistical significance (chi-squared = 3.701, P = 0.054). Similarly, no correlation between dose and stent restenosis was found. However, all stent restenosis occurred in patients receiving ≤ 66 Gy. The incidences of stent restenosis of patients receiving ≤ 66 Gy or > 66 Gy were 14.3% (5/35) and 0% (0/15), respectively (chi-squared = 2.381, P = 0.123).

## Discussions

Clarifying the dose distribution characteristics of RIBS and differences in doses calculated/measured by various methods can guide the clinical application of RIBS more effectively. As early as 1995, the AAPM TG-43 report established detailed I-125 dosimetry parameters and proposed evaluating methods for the dosimetry parameters of radioactive sources (9). As a random sampling statistical method, MC can simulate the transport of each incident particle in human tissue and, on this basis, calculate the three-dimensional dose deposition after irradiation with an accuracy close to that of reality (16). MC simulation has been widely applied to studies on radioactive source dosimetry owing to its ability to accurately model the physical process of radiotherapy (17–19).

In this study, dose distributions of different RIBSs were determined by a combination of TPS calculations, TLD measurements, and MC simulations. Despite the statistical differences among the three methods, the absolute dose difference was small (< 5 Gy), which was likely because of the large sample size (4 different specifications of stents and 6 seed activities) and a consistent trend. Values calculated by the TPS lay in the middle of the three methods (higher than TLD measurements but lower than MC simulations) and were less than 5% different from to those simulated by MC. In contrast, TLD measurements demonstrated the largest deviation (a maximum of 11.7%), which was predominantly caused by the superposition of doses from multiple radioactive seeds and the rapid dose fall-off around the RIBS. In general, TPS calculations showed good clinical accuracy and an acceptable dose deviation. When analyzing the effects of the length, diameter, and seed spacing of the stent on dose distribution, stent length and seed spacing was seen to exert substantial effects on the dose. More specifically, the longer the stent, the higher the dose, which was again caused by superposition of doses from multiple radioactive seeds. In addition, as the isodose lines produced by a seed spacing of 1.0 cm had better conformability and uniformity, in clinical practice, it was preferred to arrange the seeds at an interval of 1.0 cm. The recommended seed activity of a single I-125 seed for the treatment of esophageal cancer was 0.4–0.8 mCi based on *in vitro* and *in vivo* experiments (20). In our study, TPS isodose lines showed multiple cold spots when the activity was in the range of 0.4–0.5 mCi, whereas higher activities could lead to an increased risk of radiotoxicity. Therefore, most RIBS treatment was performed at a seed activity of 0.6 mCi in clinical practice.

Studies have shown vastly different therapeutic effects of RIBS in treating esophageal cancer, with the median survival ranging from 4 to 11 months. On the contrary, reports on the survival of patients treated with conventional stents are more consistent, ranging from 3 to 5 months (7, 8, 21–23). In terms of the relief of dysphagia and the incidence of complications, the performance of seed stents is similar to that of conventional stents (P > 0.05) (8, 24). In a randomized controlled study conducted by Zhu et al. (8) that included 148 patients (73 in the RIBS group and 75 in the conventional stent group), incidences of common complications, including severe chest pain (23% versus 20%), fistula (6% versus 7%), pneumonia (15% versus 19%), bleeding (7% versu 7%), and recurrence of dysphagia (28% versus 27%), were not significantly different. In our study, patients’ survival was relatively short (median survival was 4.4 months). Among all complications, the incidence of moderate to severe pain (20%), perforation (6%), and pneumonia (10%) was of a similar level, while that of bleeding was high (28%). The poor survival and the high incidence of fatal complications observed in this study were related to the fact that most patients were either relapsed/uncontrolled patients (62%) or had previously received radiotherapy (58%). In particular, patients with a previous history of radiotherapy showed low survival rates and high incidences of fatal complications. In addition, differences were statistically significant when compared these patients with those of patients without a history of radiotherapy (P = 0.021 and 0.019, respectively). This finding is consistent in reports by Zhu and Liu (8, 23), who listed previous history of radiotherapy as a poor prognostic factor. Since the incidence of fatal complications was substantially higher in patients with a history of radiotherapy than in those without, it was considered that the poor survival of the study mainly originated from complications. In particular, gastrointestinal bleeding is a complication that requires particular attention in patients whose interval of stent and radiotherapy was less than 6 months. Although the prognosis of patients who relapse after radiotherapy is even poorer, due to extremely limited treatment options, whether stent implant should be performed requires a joint decision made by the physician, patient, and patient’s family after carefully weighing the pros and cons. This study observed a low incidence of restenosis (8%) compared to a rate of 12.3% and 13.8% reported in literatures adopting conventional stents (25, 26). This is likely because of the superior dose distribution created by the more reasonable seed arrangement in the RIBS, which plays an important role in the prevention and treatment of restenosis caused by tumor overgrowth or in-growth. Stent restenosis occurred at a median interval of 108 days (31–196 days) after RIBS implanted in our study, by comparison, the median time of restenosis of conventional metal stents was about 2-30 weeks, which was related to tumor overgrowth, stent migration, granulation hyperplasia, food bolus obstruction and so on (25, 27). This also suggests that RIBS could probably delay the occurrence of restenosis, but due to the small number of cases and large time span, further confirmation is needed. Despite the short survival of patients receiving RIBS treatment, in actual clinical practice, most patients who need stent implant are advanced or relapsed or are refractory patients who are no longer eligible for surgery or chemoradiotherapy and yet suffer from severe obstructive symptoms. Therefore, treatment should focus on relieving symptoms and improving the quality of life. For these patients, RIBS can extend their survival by 4.4 months and allow them to eat during this period, making it valuable for palliative care. However, the risk of complications associated with the technique should be fully described to patients and their families prior to the treatment.

Dosimetric analysis in this study did not discover any factors affecting patients’ survival or incidence of complications. Potential indicative factors included D90 ≤ 50 Gy or > 50 Gy, which resulted in respective incidences of fatal complications of 18.8% and 47.1% with a difference close to statistical significance (P = 0.054); and D90 ≤ 66 Gy or > 66 Gy, which resulted in incidences of 14.3% and 0% (P = 0.123) of stent restenosis, respectively. Since existing dose evaluation methods for RIBS are still not standardized, only D90 was adopted in this study. In addition, due to large patient heterogeneity and incomplete information on patients’ tumor conditions and previous radiation doses, subsequent prospective research with more appropriate indicators is required to further clarify the role of RIBS in the treatment of esophageal cancer.

The limitations of the study are as follows: ① The analysis of dosimetry was elementary, especially without considering the influence of tissue heterogeneity and esophageal cavity on dosimetry, and solutions should be developed in next research. ② Retrospective study, follow-up data may be inaccurate or bias; ③ Because the results of survival and morbidity were not prominent, the significance of dosimetric data analysis was limited; ④ Because most patients were advanced, recurrent and refractory tumors, with poor prognosis and short survival time, it was difficult to observe long-term efficacy and complications; ⑤ The study had a high incidence of complications, which may need to be further refined in terms of technology, methods and patient selection, so as to benefit patients more specifically.

## Conclusion

Doses around the RIBS calculated by the TPS lay between those measured by the TLD and those simulated by the MC, indicating that that TPS calculations are suitable for clinical applications. In addition, the overall absolute dose differences among the three methods were small. Dose distribution was affected by seed activity, seed spacing, and stent length. Most RIBS treatment in this study was carried out with a seed spacing of 1.0 cm and a seed activity of 0.6 mCi. The application of RIBS in treating severely obstructed patients with esophageal cancer can effectively alleviate obstructive symptoms, but with a relatively a high incidence of fatal complications. Relevant research should further identify the people who can benefit from the RIBS and focus on how to improve the safety and effectiveness of RIBS in the treatment of esophageal cancer.

## Data Availability Statement

The original contributions presented in the study are included in the article/supplementary material. Further inquiries can be directed to the corresponding authors.

## Ethics Statement

Ethical review and approval was not required for the study on human participants in accordance with the local legislation and institutional requirements. Written informed consent was not provided because this is a retrospective study which only analyzed the data of previously treated patients retrospectively. The patient signed the informed consent form before stent implantation, but it did not involve the informed consent form for the study.

## Author Contributions

Conceptualization, JW and KZ. Methodology, ZJ and JW. Soft-ware, ZJ and QY. Validation, LL, CX, and XZ. Formal analysis, ZJ and QY. Investigation, SY and YJ. Resources, LL and HS. Data curation, LL, QY, CX, and XZ. Writing—original draft preparation, ZJ and QY. Writing—review and editing, JW and KZ. Visualization, ZJ. Supervision, JW. Project administration, JW and KZ. ZJ and QY contributed equally to this work, so they are listed as co-first authors. All authors contributed to the article and approved the submitted version.

## Conflict of Interest

The authors declare that the research was conducted in the absence of any commercial or financial relationships that could be construed as a potential conflict of interest

## Publisher’s Note

All claims expressed in this article are solely those of the authors and do not necessarily represent those of their affiliated organizations, or those of the publisher, the editors and the reviewers. Any product that may be evaluated in this article, or claim that may be made by its manufacturer, is not guaranteed or endorsed by the publisher.
